# Update on Endometrial Stromal Tumours of the Uterus

**DOI:** 10.3390/diagnostics11030429

**Published:** 2021-03-03

**Authors:** Iolia Akaev, Chit Cheng Yeoh, Siavash Rahimi

**Affiliations:** 1 School of Pharmacy and Biomedical Sciences, University of Portsmouth, St. Michaels Building, White Swan Road, Portsmouth PO1 2DT, UK; rahimi.siavash@gmail.com; 2 Department of Oncology, Portsmouth Hospitals University NHS Trust, Southwick Hill Road, Portsmouth PO6 3LY, UK; ChitCheng.Yeoh@porthosp.nhs.uk; 3 Department of Histopathology, Brighton and Sussex University Hospitals NHS Trust, Royal Sussex County Hospital, Brighton BN2 5BE, UK

**Keywords:** endometrial stromal sarcoma, uterine sarcoma, ESN, ESS, EST, LG-ESS, HG-ESS, *YWHAE-NUTM2*, *ZC3H7B-BCOR*, NTRK-uterine tumours, UUS

## Abstract

Endometrial stromal tumours (ESTs) are rare, intriguing uterine mesenchymal neoplasms with variegated histopathological, immunohistochemical and molecular characteristics. Morphologically, ESTs resemble endometrial stromal cells in the proliferative phase of the menstrual cycle. In 1966 Norris and Taylor classified ESTs into benign and malignant categories according to the mitotic count. In the most recent classification by the WHO (2020), ESTs have been divided into four categories: Endometrial Stromal Nodules (ESNs), Low-Grade Endometrial Stromal Sarcomas (LG-ESSs), High-Grade Endometrial Stromal Sarcomas (HG-ESSs) and Undifferentiated Uterine Sarcomas (UUSs). ESNs are clinically benign. LG-ESSs are tumours of low malignant potential, often with indolent clinical behaviour, with some cases presented with a late recurrence after hysterectomy. HG-ESSs are tumours of high malignant potential with more aggressive clinical outcome. UUSs show high-grade morphological features with very aggressive clinical behavior. With the advent of molecular techniques, the morphological classification of ESTs can be integrated with molecular findings in enhanced classification of these tumours. In the future, the morphological and immunohistochemical features correlated with molecular categorisation of ESTs, will become a robust means to plan therapeutic decisions, especially in recurrences and metastatic disease. In this review, we summarise the morphological, immunohistochemical and molecular features of ESTs with particular reference to the most recent molecular findings.

## 1. Introduction

Endometrial stromal tumours (ESTs) are a rare, fascinating and complex subset of mesenchymal uterine neoplasms with heterogeneous morphological, immunohistochemical and genetic features. ESTs constitute ∼10% of uterine mesenchymal tumours [1].

Approximately 50% of endometrial stromal sarcomas (ESSs) occur in premenopausal women and the majority is detected at stage I of the International Federation of Gynecology and Obstetrics (FIGO) [2].

Morphologically, ESTs resemble endometrial stromal cells in the proliferative phase of the menstrual cycle. In 1966 Norris and Taylor attempted to classify ESTs in their seminal manuscript [3].

They divided the lesions into two groups; the first group with pushing margins was labelled as stromal nodules and the second group with infiltrating margins was defined as endolymphatic stromal myosis or stromal sarcoma according to the mitotic index: lesions with ≤ 10 mitoses per 10 HPF (high-power field) were classified as endolymphatic stromal myosis and neoplasms with ≥ 10 mitoses per 10 HPF were categorised as stromal sarcomas. In view of the clinical outcome (100% survival rate within five years), stromal nodules were considered benign. Patients with stromal sarcoma presented with 55% survival rate within five years. The authors stated that the “size of the primary tumor and presence of vein invasion showed a slight correlation with the patient’s prognosis but no correlation was found with increasing degrees of cellular atypism” [3].

In 1982Evans showed that the prognosis of ESSs is determined by nuclear atypia/pleomorphism rather than by the mitotic rate [4].

Since the fundamental description of ESTs by Norris and Taylor, the classification of ESSs has undergone several modifications [5,6].

The last classification of the World Health Organization (WHO) in 2020 sub-categorised ESTs into four groups: Endometrial Stromal Nodule (ESN), Low-Grade Endometrial Stromal Sarcoma (LG-ESS), High-Grade Endometrial Stromal Sarcoma (HG-ESS), and Undifferentiated Uterine Sarcoma (UUS) (Table 1) [7]. Molecular analysis of ESTs has resulted in better characterisation of these tumours, this, in turn, has caused the decrease in the diagnosis of UUS, which, at present, is a heterogeneous group of tumours, as well as the diagnosis of exclusion. Similarly, NTRK-sarcomas, discovered by molecular analysis, appear to fall into the HG-ESSs. However, their endometrial stromal origin has not been established with certainty and our current knowledge fails to classify these tumours appropriately.

The aim of this review is to shed some light on the complex classification of ESTs, highlighting the varied histopathological, immunohistochemical and molecular features for each sub-group.

## 2. Endometrial Stromal Nodule (ESN) and Low-Grade Endometrial Stromal Sarcoma (LG-ESS)

ESN is a benign, whereas LG-ESS is a malignant neoplasm of the uterus (affecting the body of the uterus more than the cervix) and extra-uterine sites [8,9]. The mean age for LG-ESS is 52 years, ranging between 16 and 83 years [10]. The risk factors are pelvic radiation and prolonged use of tamoxifen or oestrogen. The most common findings are abnormal uterine bleeding and pelvic pain [11,12,13]. Other symptoms of patients with LG-ESS are uterine mass and metastases to the adnexae, lymph nodes and lungs [14].

### 2.1. ESN Morphology

**Figure 1:**
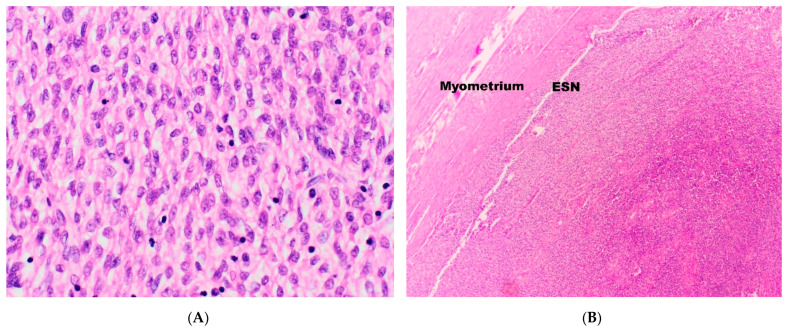
(**A**) Neoplastic cells in Endometrial Stromal Nodules (ESN) and Low-Grade Endometrial Stromal Sarcomas (LG-ESS) resemble endometrial stromal cells in the proliferative phase. (**B**) ESNs show a non-infiltrative margin.

ESNs are a proliferation of bland, uniform cells with oval nuclei and scanty cytoplasm, resembling endometrial stromal cells in the proliferative phase of the menstrual cycle (Figure 1A). ESNs exhibit prominent arterioles and well-circumscribed expansive (non-infiltrative) margins (Figure 1B). It may occasionally present with infiltrative margins, however these should be 3 mm or less in maximum dimension and not exceed three foci [15,16].

The mitotic rate is not high (less than 10 × 10 HPF). Areas of coagulative necrosis and sex-cord-like differentiation may be identified, but, by definition, lympho-vascular invasion (LVIS) is not present. Areas of smooth muscle metaplasia may be present and these should not mislead to an incorrect diagnosis of myometrial invasion. The differential diagnosis includes cellular leiomyoma and LG-ESS. The vascular pattern of ESN, composed of typical arterioles, is not a prominent feature of cellular leiomyoma. The presence of large blood vessels, one of the characteristics of cellular leiomyoma, may also be detected in ESN, but is not as conspicuous as in cellular leiomyoma. In addition, ESN usually does not contain the clefts frequently seen in cellular leiomyoma [17].

ESNs can be differentiated from LG-ESS exclusively by the presence of pushing margins and lack of LVIS [3,15,16], therefore, the definitive diagnosis of ESN can be rendered on resection specimens only and cannot be confidently established on biopsy or tissue removal systems (i.e., MyoSure resections). Nevertheless, even in resection specimens, the differential diagnosis between ESNs and LG-ESS may not be straightforward and extensive sampling may be required.

### 2.2. LG-ESS Morphology

**Figure 2:**
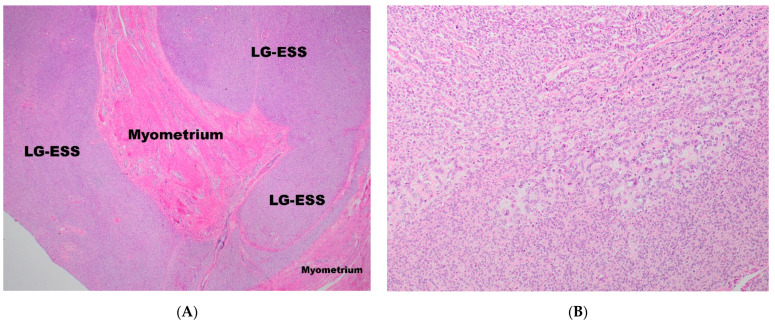
(**A**) The infiltrative, tongue-like margin in LG-ESS. (**B**) Sex-cord-like structures in LG-ESS.

LG-ESS shows the same histopathological features as ESN, except, as has already been mentioned, for the presence of infiltrative/permeative, tongue-like margins (Figure 2A) and LVIS that may also be observed in the parametrial veins [15]. 

From the purely morphological point of view, some low-grade ESTs show “hybrid” features between ESN and LG-ESS. These lesions have been labelled as ESTs with limited infiltration (EST-LI). The extent of myometrial invasion in this entity is less than in LG-ESS but more than in ESN (occasional finger-like projections into the myometrium of up to 3 mm are allowable) [17]. Obviously, the diagnosis of this lesion is highly subjective and depends on the extent of the sampling; therefore, the diagnostic reproduceable value is limited. In addition, the clinical outcome among patients with this sub-category of morphologically low-grade EST is not clear [17,18] and a small percentage of EST-LI can show malignant behaviour with distant metastases. Based on their findings Moore & McCluggage recommended in a recent paper that these neoplasms should be regarded as LG-ESS [19]. 

In some instances, the histological diagnosis of EST could be problematic, especially on biopsy. Some samples may contain foamy histiocytes, foci of hyalinisation [15,16,20], smooth muscle differentiation [17,21,22,23,24,25,26,27,28,29,30] where radiated collagen fibres of the smooth muscle component create a starburst appearance, commonly referred to as ‘starburst differentiation’ [31]. In addition, skeletal muscle differentiation [26,28,29], adipocytic metaplasia [26], rhabdoid changes [28,32,33], presence of osteoclast-like cells [27], cells with bizarre nuclei [26], cells with clear cytoplasm [34] and myxoid/fibro-myxoid changes may be present [17,25,32,35,36,37,38]. ESTs may show epithelioid morphology [39] with the presence of endometrial-type glands [34,40,41,42], pseudo-papillae [43] and sex-cord-like structures (Figure 2B) [17,24,28,29,32,44,45,46,47,48] that may present positive immunostaining with inhibin, calretinin, CD99, Melan-A and Wilms tumour 1 (WT1) [7,49,50,51].

The differential diagnosis of LG-ESS includes HG-ESS, gland-poor adenomyosis, cellular leiomyoma, intravascular leiomyomatosis, leiomyosarcoma with extensive intravascular component, uterine tumours resembling ovarian sex-cord tumour (UTROSCT), adenosarcoma and perivascular epithelioid cell tumour (PECOMA) [52].

### 2.3. Immunohistochemistry

ESNs commonly demonstrate positive immunoreactivity with CD10, oestrogen receptor (ER) (ER alpha—ERα), CD56, smooth muscle actin (SMA) and vimentin. Focal positivity is observed with progesterone receptor (PR), pan-cytokeratin (AE1/3), and desmin. ESN is usually negative with CD34, CD117 (c-kit), Cyclin D1, epithelial membrane antigen (EMA), S100, WT1 and β-catenin. p53 demonstrates a wild-type pattern of expression. The mitotic rate, evaluated by Ki67 expression, is low [53].

**Figure 3:**
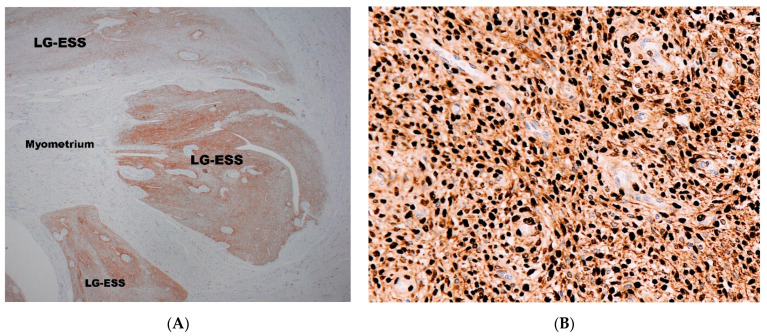
(**A**) Positive immunostaining with CD10 in LG-ESS; the surrounding myometrium is negative. (**B**) Diffuse strong nuclear immunoreactivity with ER in LG-ESS.

CD10 antibody is routinely used for the diagnosis of ESTs, (Figure 3A) and is the most popular antibody used to differentiate LG-ESS from HG-ESSs [54,55,56,57,58]. However, it is well known that CD10 is not specific for the diagnosis of ESTs [59,60] and some ESTs may show negative immunostaining with CD10 [61,62]. In addition, CD10 can also be strongly positive in undifferentiated uterine sarcoma [63].

LG-ESSs demonstrate strong expression with ERα (Figure 3B) [54,56,57,58,61,64,65,66,67,68], while ER-beta (ERβ) expression is mainly negative with occasional reported cases showing weak positivity [64]. PR expression positivity has been reported in the majority of cases (>70%), with strong positivity observed in >50% of cases [58,64,69,70] and its positivity is part of the immunohistochemical confirmation of the diagnosis of LG-ESS [54,56,61,65,66,67].

Androgen receptor (AR) expression is observed in the majority of cases [58,64]. It is worth mentioning that AR-positive immunostaining has been observed in LG-ESS harbouring *JAZF1-SUZ12* fusion and *JAZF1* rearrangement [56,69].

**Figure 4:**
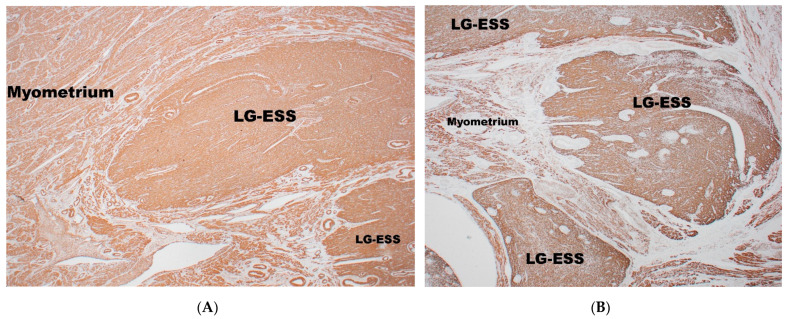
Smooth muscle actin (SMA)-positivity (**A**) and desmin-positivity (**B**) in LG-ESS.

A diffuse or focal positivity for SMA [56,58,66] (Figure 4A) and desmin (Figure 4B) is present [54,58,65,70]. H-caldesmon can be focally positive [56] and, typically, strong positivity is seen in areas of smooth muscle differentiation with a ‘starburst’ appearance [31]. A weak expression of gonadotropin-releasing hormone receptor (GnRH-R) has been reported in ~95% of cases [64]. Aromatase (CYP19A1) expression has been observed in ~85% of cases [64]. Interferon-induced transmembrane protein-1 (IFITM1), a novel marker for endometrial stromal cells, is positive in ~80% of cases. IFITM1 is superior to CD10 in differentiating ESTs from smooth muscle neoplasm (~30% in cases of leiomyomas and leiomyosarcomas) [71]. However, ~90% of carcinosarcomas demonstrate positivity with this marker, but in this case the differential diagnosis with ESTs would be based on pure morphological grounds.

The nuclear expression of β-catenin was reported in 50% of cases, with 90% demonstrating nuclear expression of Lymphoid Enhancer-binding Factor 1 (LEF1). This finding is suggestive of activation of the Wnt pathway in LG-ESS [72].

LG-ESSs are mainly reported to be negative with Cyclin D1 and BCOR [73] and both markers are commonly implemented in diagnostic differential panel between LG-ESS and HG-ESS.

However, in some cases, Cyclin D1 can be positive in the LG-ESS, but with concomitant strong ER and PR positivity and focal CD10 expression, which is unusual in high-grade endometrial stromal sarcoma [68,74]. The Ki67 proliferation index is usually low and has been reported to be positive in ~5–20% of the lesional cells [54,68,73,74]. It is worth mentioning that Ki67 can vary between tumours found in the same uterine specimen. Fujiishi et al. found the difference in the expression of Ki67 between the right anterior, right posterior, and fundal tumours to be 10%, 10%, and 3%, respectively [56].

Bcl-2 and vimentin may show immunopositivity [54,55,66]. CD34 is mainly negative [58], but some cases showed focal positivity [73,75]. Generally, there is positive immunoreactivity with WT1 [56,58,67,73], and Forkhead box protein L2 (FOXL2) has been reported to be positive in 87% of cases [58]. The data on expression of cytokeratins are conflictual. Positive immunoreactivity to AE1/3 and CAM5.2 has been reported [52,53], but negative cases have also been described [67,73].

INI-1 (SMARCB1, hSNF5, BAF47) expression seems to be retained [73]. S100, CD31, CD117 (c-kit), creatine kinase (CK), EMA, Melan-A, HMB-45, PAX8, inhibin, synaptophysin, chromogranin, DOG-1 and CD99 are usually negative [55,61,66,67,69,73,76,77].

### 2.4. Molecular Biology

**Figure 5:**
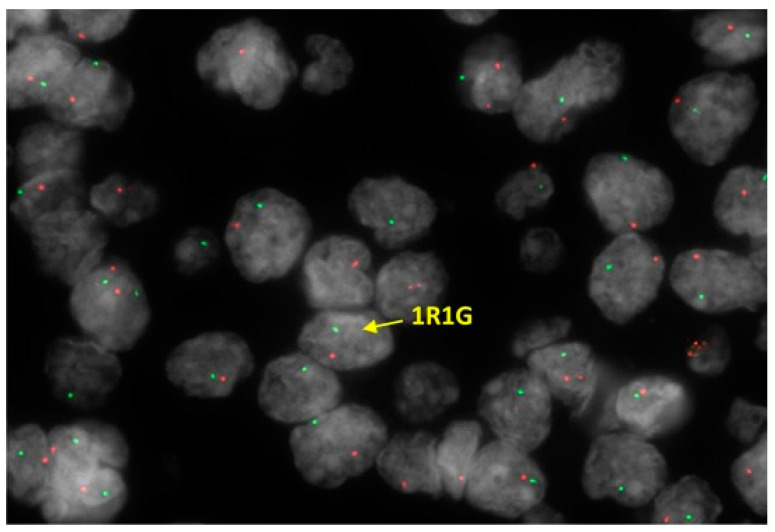
Fluorescence in situ hybridization (FISH) performed on LG-ESS, which shows *JAZF1* rearrangement using dual colour break apart probe. Additionally, the other copy of *JAZF1* is lost. Results are expressed as split R (separate red signal) and G (separate green signal) rearrangement.

LG-ESSs are genetically heterogeneous with relatively numerous identified chromosomal translocations resulting in gene fusions. However, approximately one third of these tumours do not harbour genetic fusions [7]. *JAZF1-SUZ12* is the most common gene fusion, present in approximately half of the cases [31,78,79] and related to the cytogenetic hallmark of ESN and LG-ESS (Figure 5). The minority of cases of LG-ESS display other gene fusions, including *EPC1-PHF1* [80], *MEAF6-PHF1* [81], *JAZF1-PHF1* [80], *MBTD1-CXorf67 (MBTD1-EZHIP)* [82], *BRD8-PHF1* [83], *JAZF1-BCORL1* [84], *EPC2-PHF1* [85]. Recently, *MEAF6-PHF1* has also been demonstrated in ESNs [59].

Dickson et al. reported two cases of ESSs with clinical aggressive behaviour, showing two novel *EPC1* genetic fusions: *EPC1-SUZ12* and *EPC1-BCOR* [86]. Other novel fusions include *MEAF6-SUZ12* [73], *MAGED2-PLAG1* [87] and *MBTD1-PHF1* [88].

Novel fusions that have been recently detected using RNA sequencing include KAT6B-KANSL1, RNF111-ARID2, ESR1-NCOA3, PTCH1-GLI1, SYNGAP1-JAZF1, PHF21A-NFIA, PHF21A-CETP, ACTB-GLI1 and GREB1-NCOA2 [89].

Some LG-ESSs harbouring *YWHAE-NUTM2* fusion, which is a molecular characteristic of high-grade endometrial sarcomas, have also been reported [89,90].

## 3. High-Grade Endometrial Stromal Sarcoma (HG-ESS)

On gross examination, these tumours usually show haemorrhage and necrosis [5,91,92,93]. The morphological spectrum varies according to the genetic aberrations.

### 3.1. YWHAE-NUTM2

#### 3.1.1. Morphology

**Figure 6:**
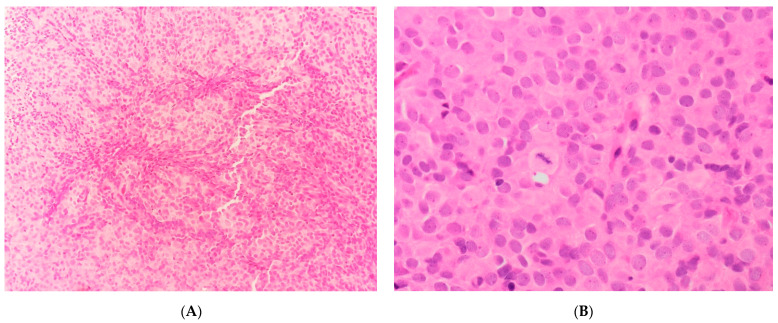
(**A**) Vague nested architecture in *YWHAE-NUTM2* sarcoma. (**B**) Cellular morphological details in *YWHAE-NUTM2* sarcoma.

These neoplasms [5,90,91,92,94] show necrosis with a permeative pattern of myometrium invasion. LVIS is a constant finding. The cellular population is a mixture of round and spindle cells. The round cell component displays high cellularity with a vague nested architecture (Figure 6A), composed of cells with a scanty/moderate eosinophilic cytoplasm (Figure 6B). The nuclei exhibit a finely granular to slightly vesicular chromatin with an irregular nuclear membrane, no prominent nucleoli and no significant nuclear atypia/pleomorphism. Sex-cord–like differentiation and pseudo-glandular/pseudo-papillary pattern may rarely be observed. The mitotic rate within the round cells component is high.

**Figure 7:**
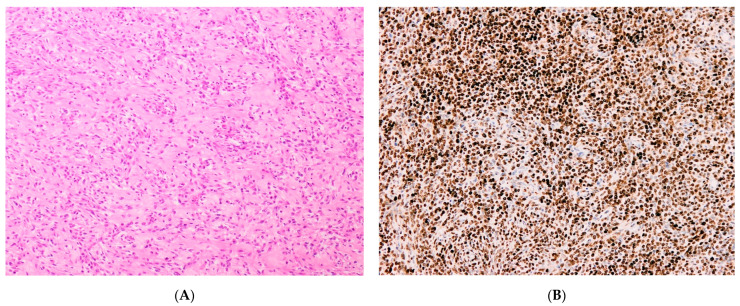
(**A**) The fibro-collagenous low-grade spindle cell component in *YWHAE-NUTM2* sarcoma. (**B**) The positive nuclear Cyclin D1 immunostaining in the round cell component of *YWHAE-NUTM2* sarcoma.

Approximately 50% of cases show a low-grade spindle cell component (Figure 7A) with low/intermediate cellularity, a fascicular pattern of growth consisting of a proliferation of bland spindle cells set in a fibro-collagenous to fibro-myxoid matrix, juxtaposed to the round cell component. The nuclei show even chromatin with no prominent nucleoli. The mitotic index in this component is low.

#### 3.1.2. Immunohistochemistry

The round cell component is positive for Cyclin D1 (Figure 7B), BCOR, CD117 (c-kit), CD56, and CD99, whereas DOG1 is negative [7,57,63,77,95,96]. Generally, ER and PR are negative, but positive staining has also been reported; CD10 shows variable expression, either positive or negative [7,56,97].

The low-grade spindle cell component may display positive staining with ER, PR, and CD10 [63,74] or show diffuse positivity with Cyclin D1 and PR and focal positivity for CD10 and p16 [74], or demonstrate positive staining with Cyclin D1 and negative staining with CD10 [96].

BCOR may show variable positivity [77]. There is no immunoreactivity with AE1/3, EMA, SMA, desmin, calretinin and inhibin [74].

#### 3.1.3. Molecular Biology

**Figure 8:**
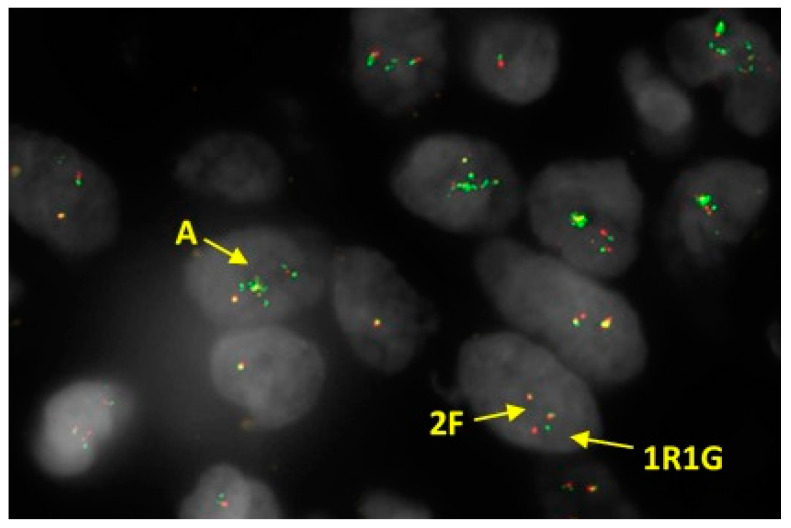
Fluorescence In Situ Hybridisation (FISH) performed on HG-ESS, which shows *YWHAE* rearrangement with amplification of the fusion signal (2F) plus additional 5’ signals (A) using dual colour break apart probe. Results expressed as F (fusion signal, i.e., normal), split R (separate red signal) and G (separate green signal) rearrangement.

These neoplasms demonstrate rearrangement of *YWHAE* (Figure 8) and commonly harbour *YWHAE-NUTM2* fusion [5,7,57]. *NUTM2* has been previously named as *FAM22* by HUGO Gene Nomenclature Committee (HGNC) Symbols [98]. There are several members of paralog genes in this family, of which *NUTM2A, NYTM2B* and *NUTM2E* protein coding genes have been associated with HG-ESSs. Mainly *NUTM2A* and *NUTM2B* have been reported to form fusion with *YWHAE* as alternative gene fusion partners [7,97]. In a more recent report, *NUTM2E* (paralog of *NUTM2B*) has also been detected as an alternative gene fusion partner [89].

*EPC1-BCOR, EPC1-SUZ12* and *BRD8-PHF1* fusions have also been reported, but they are rare [63,86]. A few cases have demonstrated a nucleotide variation with mutation in *BCOR* (NM_017745 (*BCOR* exon 4):c.2570_2571del (p.E857fs) and in *BCORL1* (NM_021946 (*BCORL1* exon 7):c.A4256T (p.K1419I) [89], in addition to the presence of *YWHAE-NUTM2E* fusion.

*WT1* gene expression is often absent or shows low expression levels [89]. Notably, it has been previously highlighted that high immunohistochemical expression of CD117 (c-kit) can be frequently found in tumours with *YWHAE* genetic rearrangement, but c-kit-immunoreactive *YWHAE-NUTM2A/B* sarcomas have not demonstrated known mutations in *KIT* gene [99].

### 3.2. ZC3H7B-BCOR

#### 3.2.1. Morphology

These tumours often show neoplastic-type or infarct-type necrosis [92]. The pattern of myometrial invasion could be infiltrative and tongue-like, similar to LG-ESS, or may display a broad front of invasion with irregular borders. A mixed pattern may also be present. LVIS is a common finding. The neoplasm shows a fascicular pattern and is composed of cells with eosinophilic cytoplasm (scanty/moderate or abundant), and spindle/oval, occasionally round, nuclei with a finely dispersed chromatin and no discernible nucleoli. Although infrequent, severe nuclear atypia/pleomorphism has been described. The stroma is myxoid in the majority of cases and collagen plaques may be identified. The intra-tumoural vessels may be large-sized or arterioles; a hemangiopericytoma-like vascular pattern may be seen. Occasionally, tumours may contain benign-appearing endometrioid glands. The proliferation rate may be very low (1 × 10 HFP) or moderate/high (50 × 10 HFP). The accompanying LG-ESS component has not been reported.

A rare novel sub-type of HG-ESS with *ZC3H7B-BCOR* fusion has been described that shares significant histopathological overlap with myxoid leiomyosarcoma [100].

#### 3.2.2. Immunohistochemistry

There is positive staining with Cyclin D1 and CD10; immunoreactivity with BCOR is seen in ~50% of cases, but in a recent paper, the BCOR positivity was up to ~80% of the lesional cells [77,101,102]. Expression of ER and PR is variable. Focal positive staining with SMA and caldesmon can be identified, but positivity with desmin is usually negative [7]. Some recent reports also demonstrated immunoreactivity with TLE1 (Transducin-like enhancer protein 1), CD99 and BCL2 [101,102,103].

Negative immunoreactivity with cytokeratins (MNF116), SMA, desmin, h-caldesmon, myogenin, myo-D1 (myoblast determination protein 1), STAT6 (signal transducer and activator of transcription 6), CD34, SOX-10 (Transcription factor SOX-10), S100, HMB45, ER, PR, CD117 (c-kit), MDM2 (E3 ubiquitin-protein ligase Mdm2) and SYT has been reported [101,102]. Nuclear staining for INI-1 is retained [102]. Ki67 proliferation index can be demonstrated between 10–25% of neoplastic cells [103]. Recently, positive staining for Pan-Trk has been reported in some cases [7,104,105].

#### 3.2.3. Molecular Biology

*ZC3H7B-BCOR* and its reciprocal fusion are mainly associated with and reported in these neoplasms [77,93,105]. Recent reports showed *MDM2*, *FRS2* and *CDK4* amplification and loss of *CDKN2A* in some cases [89,105,106].

Yoshida et al. showed elevated expression of *BCOR* and significant upregulation of *ZIC2, HOXA13* and *NTRK3* in an extra-uterine case (chest wall) of HG-ESS with *ZC3H7B-BCOR* fusion [107].

### 3.3. BCOR Internal Tandem Duplication (ITD)

#### 3.3.1. Immunohistochemistry

Clinical experience with regard to these tumours is very limited, with only a few reported cases. They show a different immunoprofile with respect to *ZC3H7B-BCOR* neoplasms. They display diffuse positive immunoreactivity with BCOR and Cyclin D1, less positive staining for CD10 and mostly negative staining with ER and PR. In addition, they may show immunoreactivity with desmin. SMA and caldesmon seem to be negative [77,93,108]. There is strong and diffuse cytoplasmic expression of pan-Trk [109].

#### 3.3.2. Molecular Biology

Juckett et al. reported that *BCOR-ITDs* occurred most frequently in exon 15 and near C-terminus, and were present in 52.4% cases of uterine sarcomas. Interestingly, the tested cases did not carry any of the simultaneous gene fusions typically associated with ESSs [93].

Lin et al. reported no amplification of *CDK4* and/or *MDM2*; however, a homozygous deletion of *CDKN2A* and *CDKN2B* was present in 20% of cases. Mutations in *STAG2, PASK, SMARCB1, ATRX, CTNNB1* and *ARID1A* were also seen in the minority of the tested *BCOR-ITDs* cases [105]. Upregulation of the expression of *NTRK3, FGFR3, RET, BCOR*, *GLI1* and *PTCH1* genes has also been reported [109].

## 4. High-Grade Sarcomas with Uncertain Endometrial Stromal Origin

### NTRK-Uterine Tumours

#### Morphology, Immunohistochemistry and Molecular Biology

These sarcomas show no definite endometrial stromal origin. Chiang et al. [110] describes a few cases of a sub-type of sarcomas with novel *NTRK* fusion. These tumours demonstrate *RBPMS-NTRK3, TPR-NTRK1, LMNA-NTRK1* and *TPM3-NTRK1* gene fusions and seem to affect premenopausal women with frequent cervical involvement (three in cervix uteri and one in corpus uteri). They show infiltrative or expansive myometrial invasion and are composed of fascicles of cells with spindle nuclei, small nucleoli and abundant eosinophilic cytoplasm. Severe nuclear atypia/pleomorphism and necrosis may be present. The stroma is myxoid/edematous. The vascular pattern may be either delicate with thin-walled vessels or composed of thick-walled blood vessels. LVIS is not identified. The proliferation rate is relatively high. Immunohistochemistry shows positive immunostaining with CD10 (not all cases), focal positive staining with SMA and very focal positivity (<10% of lesional cells) with S100. There is positive immunoreactivity with TrkA and pan-Trk. H3K27me3 expression is retained. AE1/3, desmin, ER, PR, CD34 and SOX-10 are negative [110].

Croce et al. [111] reported a group of cervical, uterine and vaginal spindle-cell sarcomas displaying morphological resemblance with fibro-sarcomas. The authors divided these neoplasms into three categories:

- Cervical *NTRK* fusion-positive (*TPM3-NTRK1* and *EML4-NTRK3*) sarcomas with diffuse immunostaining with Trk;
- *COL1A1-PDGFB* fusion-positive sarcomas with diffuse positive staining with CD34;
- S100 immunoreactive sarcomas with no genetic rearrangement.

The fascinating finding is that *COL1A1-PDGFB* rearrangement is a characteristic of dermatofibrosarcoma protuberans (DFS) and has not been reported in uterine sarcomas. The authors classified the S100-positive sarcomas as malignant peripheral nerve sheath tumours. This group showed diffuse positive staining with Cyclin D1 and focal positivity with BCOR. CD34, HMB45, Melan A, ER, PR, desmin, Trk and H3K27me3 were negative [111].

Grindstaff et al. recently described an additional *COL1A1-PDGFB* fusion-positive uterine case with diffuse CD34 immunopositivity. There was focal positive staining with CD10, SMA and positive aberrant expression of p53. The Ki67 proliferation index was high. Desmin, caldesmon and p16 were negative [112]. 

In a uterine sarcoma with *TMP3-NTRK1* rearrangement, described by Boyle et al., cytokeratins, SMA, desmin, ER, PR, SOX-10, caldesmon, ALK1, CD117 (c-kit), DOG1, CD21 and CD23 were tested and were all negative. CD10 and Pan-Trk were positive. CD34 displayed a weak cytoplasmic staining and Cyclin D1 was focally positive [113].

Michal et al. described a *STRN-NTRK3*-rearranged uterine sarcoma with a peculiar morphology. The neoplasm contained bland epithelioid/plasmacytoid cells embedded in a myxoid stroma with a complex, arborising vascular pattern and focal peri-vascular hyalinisation. There was infarct-type necrosis but frank tumoural necrosis, significant nuclear pleomorphism and mitoses were not seen. The neoplastic cells displayed strong positive immunostaining with S100, CD34 and nuclear and cytoplasmic Pan-Trk. The extensive immunohistochemistry with keratins, CD10, myogenic, peri-neural/neural, melanocytic, neuroendocrine and vascular markers showed negative staining. The Ki67 proliferation rate was low [114].

## 5. High-Grade Sarcoma Not Otherwise Specified (NOS)

The 2020 WHO classification of EST contains a vague sub-category that seems to be associated with a LG-ESS component [7].

## 6. Undifferentiated Uterine Sarcomas (UUS)

### UUS

#### Morphology, Immunohistochemistry and Molecular Biology

UUS is a rare uterine sarcoma [115,116,117] that includes a variegated group of neoplasms with no specific line of differentiation and, by definition, is a diagnosis of exclusion. The patients with UUS are postmenopausal and present with uterine bleeding and pelvic pain. Macroscopically, UUS usually displays necrosis and haemorrhage. Morphologically, UUS can be sub-divided into monomorphic and pleomorphic sub-types [118]. It consists of epithelioid and/or spindled cells with high mitotic rate and a destructive pattern of myometrial invasion. LVIS and necrosis are common findings [119]. When UUS consists of a uniform cellular population, HG-ESS harbouring *YWHAE-NUTM2 (FAM22)* fusion should be ruled out. Conversely, some pleomorphic sarcomas may exhibit *YWHAE, JAZF1* and *NTRK* rearrangements. Therefore, these neoplasms should not be classified as UUS [77,78,110,111]. Tumours displaying high-grade nuclear atypia/pleomorphism, but associated with a LG-ESS component, should be classified under endometrial stromal sarcoma NOS category [118].

UUS usually shows positive staining with p16, with positive aberrant p53 expression. Focal positive immunostaining with CD10, PR, Cyclin D1 and β-catenin may be present [74,119].

Cotzia et al. showed that some UUS could be under recognised HG-ESSs because of positive immunostaining with BCOR, Cyclin D1, CD10, ER and PR [63].

*GREB1-NCOA2*-uterine tumour is a high-grade endometrial sarcoma with a novel *GREB1-NCOA2* fusion, consisting of spindle/polygonal cells with high-grade nuclear atypia. The neoplasm shows a high mitotic rate, necrosis, LVIS and invasion of the cervix and both parametria [120]. Immunohistochemistry shows that the lesional cells positive for vimentin, AE1/3, ER and PR and express desmin weakly. CK5/6, CK7, CK14, CD10, CD45, CD117, chromogranin A, synaptophysin, actin, SMA, Myf-4, HMB-45, TTF1, CD10, Ber-EP4 and PAX8 were negative. However, in view of the immunoprofile of this case, diagnosis of carcinosarcoma cannot be excluded.

UUSs can harbour simultaneous numerous gene fusions. Brahmi et al. [89] have reported cases with multiple detected (>3) in-frame fusions and a case with novel translocation *CREBBP-BCOR*. Moreover, UUS cases demonstrated complex genomic profiles with numerous gene fusions and/or had mutations involving *TP53, KRAS, NRAS* or *BRAF* genes.

Novel *YWHAE* gene rearrangement with no known partner has been reported in two UUSs with marked nuclear pleomorphism. This novel rearrangement may represent novel fusions in this sarcoma subtype [63]. A single case (1/23) harbouring *HMGA2-RAD51B* fusion has been described, which also demonstrated high expression of *NTRK3, FGFR3, RET, BCOR, GLI1* and *PTCH1,* and low expression of *ESR1*. [109]. Other cases have demonstrated low expression of these genes along with low *ESR1* (17/23) expression; however, no fusions have been observed [109].

## 7. Conclusions

In the era of Next Generation Sequencing (NGS) there is a huge effort to integrate the morphological and immunohistochemical classification of ESTs with molecular sub-categorisation. The aim of the molecular classification of ESTs, as in other neoplasms, is not a purely academic exercise, but rather an understanding of the molecular bases in order to develop a specific target therapy for each sub-category. This will be of paramount importance for planning a therapeutic strategy in the metastatic disease. Therefore, in the future, we will be witnessing discoveries of new entities and, gradually, the histopathological classification will be replaced by molecular classification. Genetic profile of ESSs with the most frequently reported and novel molecular alterations are presented in Table 2.

## Figures and Tables

**Table 1:** The classification of Endometrial Stromal Tumours defined in the fifth edition of WHO Classification of Female Genital Tumours.

Category	ICD-11 ^1^ Coding (Histopathology)	ICD-O ^2^ Coding
Endometrial Stromal Nodule	XH8C13	8930/0
Low-Grade Endometrial Stromal Sarcoma	XH1S94	8931/3
High-Grade Endometrial Stromal Sarcoma	XH2CV3	8930/3
Undifferentiated Uterine Sarcoma	XH6HY6	8805/3

^1^ ICD-11 = International Classification of Diseases, 11th revision; ^2^ ICD-O = International Classification of Diseases for Oncology.

**Table 2:** Shows molecular alterations most frequently reported in ESS.

Type	Genes Involved	Most Frequent Reported Fusions/Gene Rearrangements/Alterations	Translocations	References
LG-ESS	*JAZF1*	*MEAF6-SUZ12*	t(1;17)(p34;q11)	[73]
	*SUZ12*	*JAZF1-SUZ12*	t(7;17)(p15;q11)	[31,78,79]
	*PHF1*	*JAZF1-PHF1*	t(6;7)(p21;p15)	[80]
	*BCORL1*	*EPC1-PHF1*	t(10;6)(p11;p21)	[80]
	*EPC1*	*MEAF6-PHF1*	t(1;6)(p34;p21)	[81]
	*EPC2*	*MBTD1-CXorf67 (MBTD1-EZHIP)*	t(X;17)(p11.2;q21.33)	[82]
	*MEAF6*	*BRD8-PHF1*	t(5;6)(q31;p21)	[83]
	*MBTD1*	*JAZF1-BCORL1*	t(7;X)(p15;q26.1)	[84]
	*EZHIP*	*EPC2-PHF1*	t(2;6)(q23;p21)	[85]
	*BRD8*	*EPC1-SUZ12*	t(10;17)(p11;q11)	[86]
	*BCOR*	*EPC1-BCOR*	t(10;X)(p11;p11)	[86]
	*MAGED2*	*MAGED2-PLAG1*	t(X;8)(p11.21;q12.1)	[87]
	*PLAG1*	*MBTD1-PHF1*	t(X;6)(p11.2;p21)	[88]
	*YWHAE*	*YWHAE/NUTM2*	t(10;17)(q22;p13)	[89,90]
	*NUTM2*	*MEAF6-SUZ12*	t(5;6)(q31;p21)	[83]
HG-ESS	*YWHAE*	*YWHAE/NUTM2*	t(10;17)(q22;p13)	[5,7,57]
	*NUTM2A/B/E*	*EPC1-BCOR*	t(10;X)(p11;p11)	[63,86]
	*EPC1*	*EPC1-SUZ12*	t(10;17)(p11;q11)	[63,86]
	*SUZ12*	*BRD8-PHF1*	t(5;6)(q31;p21	[63,86]
	*BCOR*	*BCOR* alteration	none	[89]
	*BRD8*			
	*PHF1*			
ZC3H7B-BCOR HG-ESS	*ZC3H7B*	*ZC3H7B-BCOR*	t(22;X)(q13;p11)	[77,93,105]
	*BCOR*	*BCOR-ZC3H7B*	t(X;22)(p11;q13)	
BCOR ITD HG-ESS	*BCOR*	*BCOR ITD*	none	[93]
NTRK-uterine sarcomas HG-ESS	*TPR*	*TPR-NTRK1*	1q31.1-1q23.1	[110]
	*NTRK1*	*LMNA-NTRK1*	1q22-1q23.1	[110]
	*LMNA*	*TPM3-NTRK1*	1q21.3-1q23.1	[110,111]
	*TPM3*	*RBPMS-NTRK3*	t(8;15)(p12;q25.3)	[110]
	*RBPMS*	*EML4-NTRK3*	t(2;15)(p21;q25.3)	[111]
	*NTRK3*	*COL1A1-PDGFB*	t(17;22)(q21.33;q13.1)	[111,112]
	*EML4*	*STRN-NTRK3*	t(2;15)(p22.2;q25.3)	[114]
	*COL1A1*	*TPR-NTRK1*	1q31.1-1q23.1	[110]
	*PDGFB*	*LMNA-NTRK1*	1q22-1q23.1	[110]
	*STRN*	*TPM3-NTRK1*	1q21.3-1q23.1	[110,111]
UUS	*YWHAE*	*YWHAE* gene rearrangement	unknown fusion partner	[63]
	*CREBBP*	*CREBBP-BCOR*	t(16; X)(p13;p11)	[89]
	*BCOR*	*HMGA2-RAD51B*	t(12;14)(q14.3;q24.1)	[109]
	*HMGA2*	*GREB1-NCOA2*	t(2;8)(p25.1;q13.3)	[120]
	*RAD51B*			
	*GREB1*			
	*NCOA2*			

## Abbreviations

AE1/3	Pancytokeratin
AR	Androgen receptor
CC.Y.	Chit Cheng Yeoh
CK	Creatine kinase
CRS	Endometrial Stromal Sarcoma
DFS	Dermatofibrosarcoma protuberans
EMA	Epithelial membrane antigen
ER	Oestrogen receptor
ERα	Oestrogen receptor alpha
ERβ	Oestrogen receptor beta
ESN	Endometrial Stromal Nodule
EST	Endometrial stromal tumours
EST-LI	EST with limited infiltration
FIGO	International Federation of Gynecology and Obstetrics
FOXL2	Forkhead box protein L2
GnRH-R	Gonadotropin-releasing hormone receptor
HG-ESS	High-Grade Endometrial Stromal Sarcoma
HPF	High-power field
I.A.	Iolia Akaev
IFITM1	Interferon-induced transmembrane protein-1
INI-1	SMARCB1, hSNF5, BAF47
ITD	Internal Tandem Duplication
LEF1	Lymphoid Enhancer-binding Factor 1
LG-ESS	Low-Grade Endometrial Stromal Sarcoma
LVIS	Lymphovascular invasion
MDM2	E3 ubiquitin-protein ligase Mdm2
myo-D1	Myoblast determination protein 1
NGS	Next Generation Sequencing
NOS	Not Otherwise Specified
NTRK	Neurotrophic receptor tyrosine kinase
PECOMA	Perivascular epithelioid cell tumour
PR	Progesterone receptor
RNA	Ribonucleic acid
S.R.	Siavash Rahimi
SMA	Smooth muscle actin
SOX-10	Transcription factor SOX-10
STAT6	Signal transducer and activator of transcription 6
TLE1	Transducin-like enhancer protein 1
UTROSCT	Uterine tumours resembling ovarian sex-cord tumour
UUS	Undifferentiated Uterine Sarcoma
WHO	World Health Organisation
WT1	Wilms tumour 1
